# Stress-dependent phosphorylation of myocardin-related transcription factor A (MRTF-A) by the p38^MAPK^/MK2 axis

**DOI:** 10.1038/srep31219

**Published:** 2016-08-05

**Authors:** Natalia Ronkina, Juri Lafera, Alexey Kotlyarov, Matthias Gaestel

**Affiliations:** 1 Department of Biochemistry, Hannover Medical School, Hannover, Germany

## Abstract

Myocardin-related transcription factor A (MRTF-A) is a known actin-regulated transcriptional coactivator of serum response factor (SRF). Stimulation of actin polymerization activates MRTF-A by releasing it from G-actin and thus allowing it to bind to and activate SRF. Here, we compared protein phosphorylation in MK2/3-deficient cells rescued or not by ectopic expression of MK2 in two independent phosphoproteomic approaches using anisomycin-treated MEF cells and LPS-stimulated mouse macrophages, respectively. Two MRTF-A sites, Ser^351^ (corresponding to Ser^312^ in human) and Ser^371^ (Ser^333^ in human), showed significantly stronger phosphorylation (12-fold and 6-fold increase) in the cells expressing MK2. MRTF-A is phosphorylated at these sites in a stress-, but not in a mitogen-induced manner, and p38^MAPK^/MK2 catalytic activities are indispensable for this phosphorylation. MK2-mediated phosphorylation of MRTF-A at Ser^312^ and Ser^333^ was further confirmed in an *in vitro* kinase assay and using the phospho-protein kinase-D (PKD)-consensus motif antibody (anti-LXRXXpS/pT), the p38^MAPK^ inhibitor BIRB-796, MK2/3-deficient cells and MRTF-A phospho-site mutants. Unexpectedly, dimerization, subcellular localization and translocation, interaction with actin, SRF or SMAD3 and transactivating potential of MRTF-A seem to be unaffected by manipulating the p38^MAPK^/MK2-dependent phosphorylations. Hence, MRTF-A is stress-dependently phosphorylated by MK2 at Ser^312^ and Ser^333^ with so far undetected functional and physiological consequences.

The p38^MAPK^/MAPKAPK(MK)-2 and-3 signalling pathway is a major regulator of stress- and cytokine-induced post-transcriptional gene expression^1^. A further role of MK2 in stress-induced transcriptional activation of immediate early genes (IEGs) via SRF was shown recently^2^. Stress-induced IEGs represent the class I of SRF target genes which are coordinated in their expression by the ternary complex factor (TCF) subclass of Ets-type SRF-cofactors^3^. Another type of SRF cofactors belongs to the myocardin family including myocardin and myocardin-related transcription factors (MRTFs)^4^. The myocardin family of transcriptional coactivators regulates class II SRF target genes involved in muscle-specific functions, actin dynamics and cell motility^5^. TCFs and MRTFs are regulated by different, cross-talking signalling pathways^6^,^7^,^8^. Activation of the classical MAP kinase pathway through Ras, Raf, MEK and ERK results in TCFs’ phosphorylation, followed by their binding to Ets DNA recognition sites and SRF^9^. Stimulation of Rho/actin signalling activates MRTFs by releasing them from actin, thus allowing them to bind and activate SRF^10^. MRTFs and TCFs interactions with SRF are mutually exclusive enabling SRF to direct the expression of different sets of target genes depending on the stimuli and the cellular context^10^,^11^,^12^,^13^.

MRTF-A is a prominent member of the myocardin family acting as a key factor linking actin dynamics with transcriptional regulation due to the presence of a unique set of functional domains required for G-actin binding, nuclear-cytoplasmic shuttling, SRF interaction and transactivation^5^,^14^. Here, we describe the identification of MRTF-A as a substrate phosphorylated by MK2 in two parallel phosphoproteomic approaches. We analyse the stress-dependent phosphorylation of MRTF-A by p38^MAPK^/MK2 in cultured cells and *in vitro.* In addition, we investigated the role of the phosphorylations detected in the modulation of the known functional properties of MRTF-A by using phosphorylation site mutants of MRTF-A and protein kinase inhibitors.

## Results

### Identification of MRTF-A as a potential MK2 substrate

**Figure 1:**
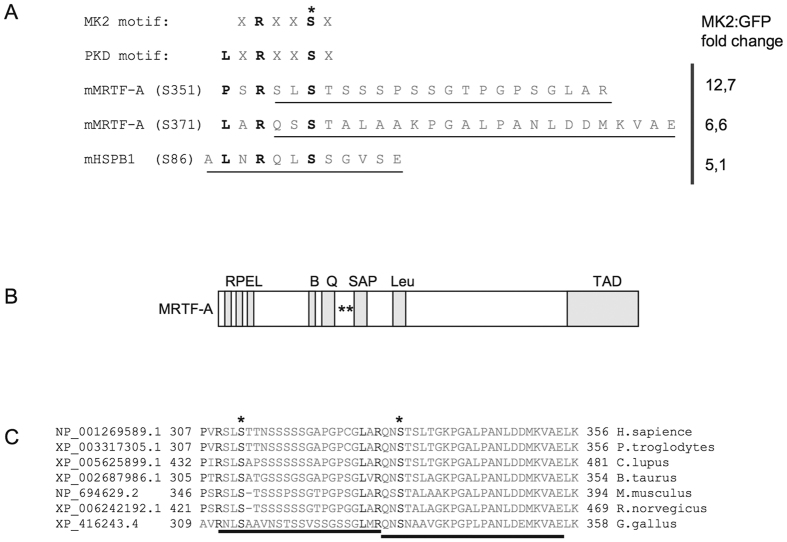
Identification of MRTF-A as a potential MK2 substrate. (**A**) MK2/3-deficient anisomycin-stimulated mouse embryonic fibroblasts rescued or not by MK2 expression were starved overnight and then stimulated with 10 μg/ml anisomycin for 30 min. Protein phosphorylation was analysed by a phosphoproteomic approach using Glu-C protease cleavage, immunoprecipitation by the phospho-Protein kinase-D (PKD)-consensus motif antibody (anti-LXRXXpS/pT) and subsequent mass spectrometry of the immunoprecipitated peptides (Cell Signaling phosphoscan). Two phosphopeptides ascribed to MRTF-A were found to be 6,6- to 12,7-fold time enriched in the MK2-rescued cells versus MK2/3 DKO cells. As a positive control the HspB1 (Hsp25) peptide showed 5.1-fold increase in MK2-rescued cells. The scheme represents comparison of identified MRTF-A phosphorylation sites with that of MK2 known substrate HspB1, consensus MK2/3 phosphorylation site and PKD-consensus motif. Matching residues are in bold, the phosphorylated S is denoted by asterix, and the peptides sequences identified in the phosphoscan are underlined. ϕ represents a hydrophobic amino acid. (**B**) Schematic representation of the MRTF-A protein. The RPEL motif, basic domain (B), Q-rich domain (Q), SAP domain, Leu zipper like domain (Leu), and transactivation domain (TAD) are shown. Sites of newly identified MK2-mediated phosphorylation (S312, S333) are indicated as asterisks. (**C**) Amino acid sequence alignment of MRTF-A from different vertebrate species. The sequences were aligned with the help of HomoloGene program from NCBI. MK2 consensus motif matching residues are in bold; the phosphorylated Serines are denoted by asterix.

Immortalized MK2/3-deficient MEFs were rescued with an retroviral coded MK2 or an empty GFP-expression vector as control to establish stable cell lines with an identical genetic background representing “wild type” (+K2) and MK2/3-knockout. These cell lines were stimulated with anisomycin, lysed and digested with GluC. The resulting peptide mixture was subjected to immunoprecipitation (IP) using antibodies against the PKD substrate consensus phospho motif (anti-LXRXXpS/pT), which is very similar to the MK2 phospho motif L/F/IXRXXpS/pT, to enrich phosphopeptides. Subsequently, a mass spectrometry analysis was performed (Cell Signalling Technology Phosphoscan) to compare the abundance of protein fragments in both probes. Using this approach the classical MK2 substrate HspB1 (Hsp25 (mouse)/Hsp27 (human)) was identified with a more than 5-fold increased abundance in the presence of MK2 validating the approach as a proper MK2 substrate screen. Two mouse MRTF-A derived peptides, 349-SLpSTSSSPSSGTPGPSGLAR-368 and 369-QSpSTALAAKPGALPANLDDMKVAE-392, were identified in this screening and their strong enrichment in the lysates from MK2-rescued MEFs as well as the presence of the MK2 consensus motif qualified MRTF-A as a potential MK2 substrate (Fig. 1A). Interestingly, peptide 349-SLpSTSSSPSSGTPGPSGLAR-368 was additionally identified in the same abundance in an independent phospho-proteomic experiment, where the phosphopeptides from LPS-stimulated MK2-deficient and MK2-rescued macrophages were compared after enrichment using antibodies against the mixed basophilic phospho motif. MK2-dependent enrichment of the same MRTF-A phosphopeptides in two independent screenings with different cell lines (MEFs, macrophages) and different stimuli (anisomycin, LPS) together with the presence of MK2 consensus motif in these peptides strongly suggests MRTF-A as a direct MK2 substrate. The potential MK2- phosphorylation sites are located between the Q-rich-domain and the putative DNA-binding SAF-A/B-Acinus-PIAS (SAP)-domain of MRTF-A and their phosphorylation might influence MRTF-A functions allosterically (Fig. 1B). The identified phosphorylation sites and their consensus motif are conserved between different vertebrate species (Fig. 1C). It should be noted that the sites S351 and S371 identified in mouse (mMRTF-A) correspond to S312 and S333 in human MRTF-A (hMRTF-A), respectively.

### p38 MAPK/MK2 activation induces MRTF-A phosphorylation

**Figure 2:**
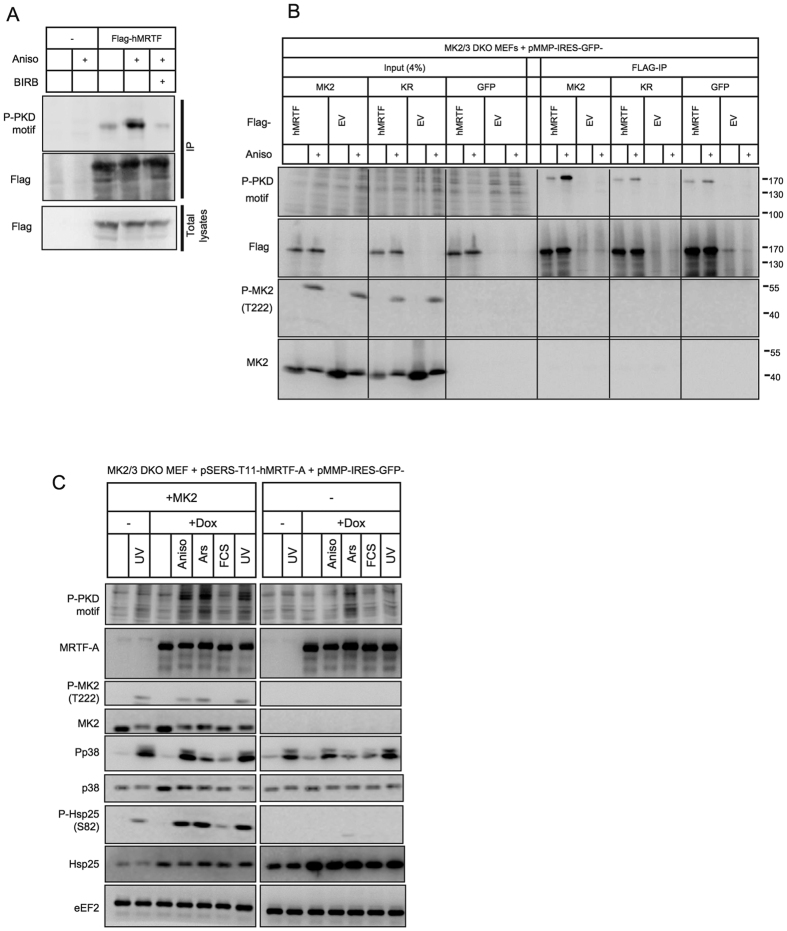
p38 MAPK/MK2 activation induces MRTF-A phosphorylation. (**A**) MRTF-A is phosphorylated in p38 MAPK dependent manner. HeLa cells transfected or not with flag-tagged hMRTF-A were starved for 5 hr and then were left unstimulated or were stimulated with 10 μg/ml anisomycin (Protein synthesis inhibitor and SAPK activator) with or without 60 min of p38MAPK inhibitor (BIRB796−1 μM) pre-treatment. Extracts were analysed directly (Total lysates panel) or following immunoprecipitation with Anti-Flag-M2 Affinity Gel (IP panel). Anti-P-PKD-motif antibody was used to detect phosphorylation of overexpressed hMRTF-A upon stress stimulation. (**B)** MK2 catalytic activity is indispensable for stress-induced hMRTF-A phosphorylation detectable by anti-P-PKD-motif antibody. MK2/3 DKO MEFs stably transduced with retroviral expression vector coding for wild type MK2 (MK2), MK2 catalytic dead-K79R-mutant (KR) or with just empty vector (GFP) were transfected by electroporation with flag-tagged-hMRTF-A (MRTF) or with empty vector (EV). After 8 hr of starvation cells were left unstimulated, or were stimulated with 1 mg/ml Anisomycin for 30 min. Extracts were analysed directly (input) or following immunoprecipitation with Anti-Flag-M2 Affinity Gel (IP). Anti-P-PKD-motif antibody were used to detect phosphorylation of overexpressed MRTF-A upon stress stimulation. (**C**) hMRTF-A phosphorylation detectable by anti-P-PKD-motif antibody is induced by different stress, but not mitogen, stimuli and is MK2-dependent. Retroviral coded human hMRTF-A in Tet-On context was introduced in MK2/3 knockout and MK2-rescued MEF cells. Overexpression of hMRTF-A was induced by 1 μg/ml doxycyclin treatment ( + Dox) for 16 hr in serum-free medium. After that cells were stimulated for 30 min with 10 μg/ml anisomycin (Anis), 200 J/qm UV-C (UV), 0,5 mM arsenite (Ars) or with 10% FCS. MRTF-A phosphorylation at S333 was monitored by anti-P-PKD-motif antibody.

MK2/3 are directly phosphorylated and exclusively activated by p38^MAPK^α,β. Therefore, we monitored the p38^MAPK^-dependence of MRTF-A phosphorylation in HeLa cells transfected with an expression construct for Flag-tagged hMRTF-A. MRTF-A detection by the PKD motif antibodies was strongly increased after anisomycin treatment and almost completely suppressed by application of the p38 inhibitor BIRB796 indicating that MRTF-A is phosphorylated upon stress in a p38-dependent manner (Fig. 2A). Anisomycin-induced MRTF-A phosphorylation was dependent on MK2 catalytic activity, since the recognition of overexpressed MRTF-A by PKD motif antibodies upon stress was reduced to almost background levels in the knockout MEFs and could not be restored by expression of the catalytic dead MK2 mutant (KR) (Fig. 2B). Next, we introduced retroviral-coded hMRTF-A in Tet-On context into MK2/3 knockout and MK2-rescued MEFs and applied stress and mitogen stimuli to define the role of MK2 in MRTF-A phosphorylation in response to different extracellular stimuli. Similar to anisomycin treatment, UV irradiation-induced MRTF-A phosphorylation was dependent on MK2 (Fig. 2C). This is consistent with our previous data that UV and anisomycin are strong activators of p38/MK2-dependent gene expression^2^. Arsenite-induced MRTF-A phosphorylation is partially MK2-dependent, while serum stimulation fails to induce the PKD motif antibodies-detectable phosphorylation of MRTF-A (Fig. 2C).

### MRTF-A is directly phosphorylated by MK2 at S312 and S333 upon stress

**Figure 3:**
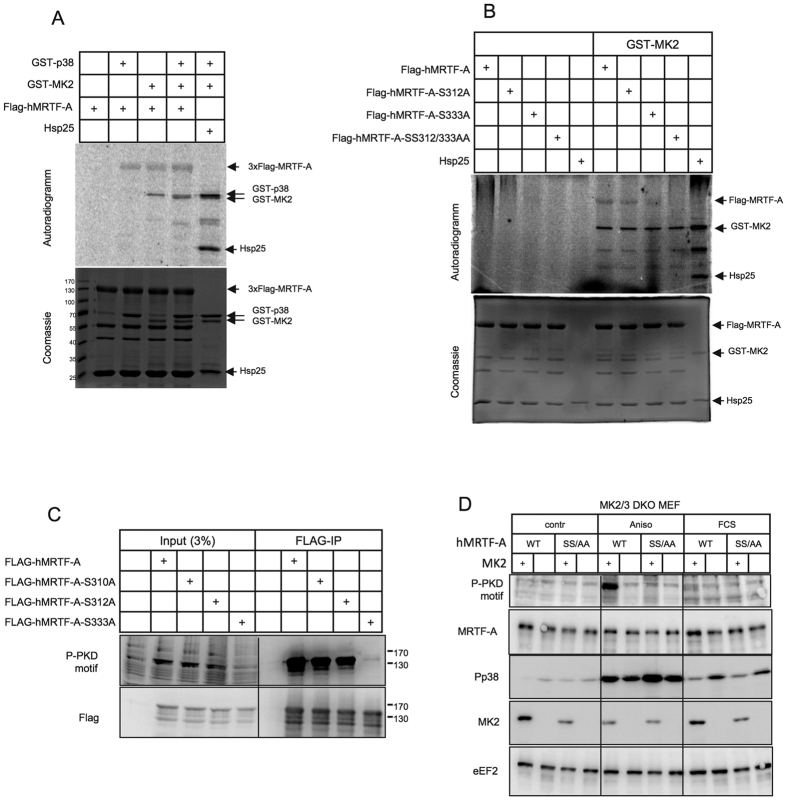
MRTF-A is directly phosphorylated by p38 and by MK2 *in vitro*. (**A**,**B)** *In vitro* kinase assay with Flag-MRTF-A as a substrate. Flag-hMRTF-A was expressed in HEK293T cells and immunoprecipitated with Anti-Flag-M2 Affinity Gel. Beads with immobilized flag-hMRTF-A were used as a substrate for *in vitro* kinase assay with bacterially purified recombinant GST-p38, GST-MK2 or both together. mHspB1 (Hsp25) is shown as a positive control. A coomassie staining and phospho-image of the reaction mixes separated in SDS-PAGE are shown. (**A**) MRTF-A is directly phosphorylated by p38 and by MK2. (**B**) MRTF-A is directly phosphorylated by MK2 at S312 and S333. Single S333A or S312A mutations were not sufficient to prevent MRTF-A phosphorylation by MK2 and only the double alanine substitution resulted in complete prevention of MK2 mediated phosphorylation. (**C**) hMRTF-A is phosphorylated at Serine 333 upon stress stimulation. HeLa cells were transfected with Flag-tagged hMRTF-A or with its different S/A mutants. After 8 hr starvation cells were stimulated with 10 μg/ml anisomycin for 30 min. Lysates were subjected to immunoprecipitation with Anti-Flag-M2 Affinity Gel and analysed in WB. All constructs were comparably expressed and immunoprecipitated (Flag panel). Mutation of Serin 333 to Alanine eliminates recognition of overexpessed hMRTF-A by anti-P-PKD-motif antibody (P-PKD motif panel). (**D**) Stress induced hMRTF-A phosphorylation at S312/S333 is MK2-dependent. MK2/3 DKO MEFs stably transduced with doxycycline-inducible hMRTF-A or hMRTF-A-SS312/333AA non-phosphorylatable mutant in the presence or absence of MK2 were treated with 1 μg/ml doxycycline in serum-free medium for 12 hr and then were left unstimulated or were stimulated for 30 min with 10% FCS or 10 μg/ml anisomycin. Cell lysates were analysed in WB with appropriate antibodies. Mutation of Ser312/S333 sites to Alanine completely prevents hMRTF-A phosphorylation detectable by the anti-P-PKD-motif antibody.

To assure the MRTF-A is a direct MK2 substrate we purified overexpressed tagged hMRTF-A from HeLa cells and performed an *in vitro* kinase assay using bacterially expressed recombinant p38^MAPK^ and MK2. Both kinases alone as well as in combination phosphorylate MRTF-A to similar extent (Fig. 3A). Single mutations S333A or S312A were not sufficient to prevent MRTF-A phosphorylation by MK2 and only the double alanine substitution resulted in complete prevention of MK2-mediated phosphorylation (Fig. 3B). p38-mediated phosphorylation of MRTF-A was not significantly influenced neither by single, nor by double mutation of S333 and S312 (Supplementary Figure 1). Hence, the data of the *in vitro* kinase assay also suggest the presence of at least one additional direct p38 phosphorylation site on MRTF-A different from S312 and S333. Mutation of the two potential MK2 phosphorylation sites revealed S333 as the major MK2-dependent phosphorylation site detected by the PKD consensus phospho motif antibodies in anisomycin-stimulated HeLa cells (Fig. 3C). Finally, we analyzed phosphorylation of hMRTF-A on Ser312 and Ser333 sites in the presence (+) or absence of MK2 (−) upon stress and mitogen activation in MEFs. Anisomycin-induced activation of p38^MAPK^ signaling resulted in strong phosphorylation of MRTF-A on the PKD consensus phospho motifs (Ser312/Ser333) mediated by MK2 (Fig. 3D). Knockout of MK2 or disruption of Ser312/Ser333 phosphorylation sites by replacement of correspondent serine to alanine (SS/AA) completely prevented the PKD phospho motif antibodies-detectable phosphorylation of MRTF-A indicating that MK2 is indispensable for stress-induced phosphorylation of MRTF-A on Ser312/Ser333. The weak phosphorylation of MRTF-A on Ser312/Ser333 in response to FCS stimulation was also MK2-dependent (Fig. 3D).

### p38/MK2 activation does not affect MRTF-A’s transactivating properties

**Figure 4:**
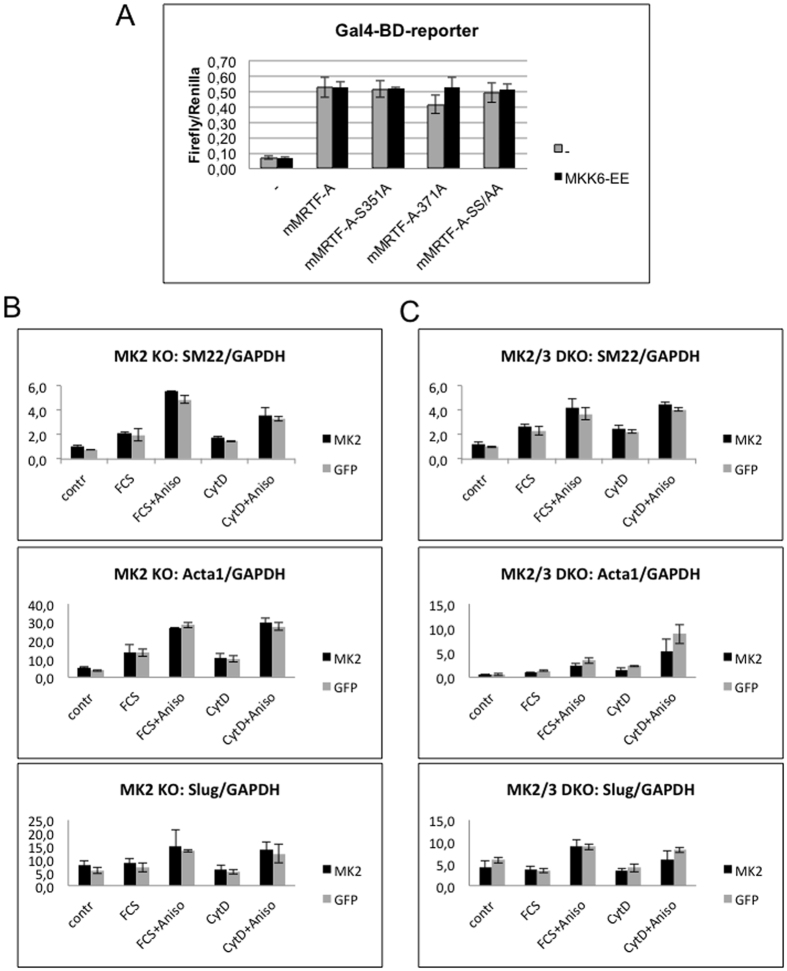
p38^MAPK^/MK2 activation does not affect MRTF-A transactivating properties. (**A**) Wild type mMRTF-A or its S351A, S371A single or double (SS/AA) mutants fused to GAL4-DNA-binding domain (Gal4-BD) were tested for transcriptional activity by using a GAL4-dependent luciferase reporter (UAS-luc) in transfected HeLa cells alone or together with constitutive active MKK6 (Flag-MKK6-EE). Normalized luciferase activity 24 h post-transfection was determined and representative data from three independent experiments performed in triplicate are presented. (**B**,**C**) Effect of serum or cytochalasin D or their combination with anisomycin on MRTF-A target gene expression in MK2 deficient (B) or MK2/3 double deficient (C) MEF cells rescued or not with MK2. Cells were starved overnight and then were left unstimulated or were stimulated for 90 min with 2 μM cytochalasin D (CytD) or 10% FCS with or without their combination with 10 μg/ml anisomycin. Relative mRNA levels for acta1, SM22 and slug were measured by quantitative real-time PCR using the SYBR green method.

Although the confirmed MK2-dependent phosphorylations in MRTF-A are located between the Q-rich and SAP-domains, it may well be that these phosphorylations determine MRTF-A functions allosterically. To assess the influence of p38^MAPK^/MK2-mediated phosphorylation of MRTF-A on its transcriptional activity we used GAL4 DNA-binding domain fused to the complete coding region of mMRTF-A or mMRTF-A-S351A, -S371A or SS351/371AA non-MK2-phosphorylatable mutants and assayed their abilities to activate a GAL4-dependent luciferase reporter in transfected HeLa cells. As a potent activator of the p38^MAPK^/MK2 pathway, the co-expression of constitutive active MKK6-EE construct as direct p38^MAPK^ activator was used. In this assay, expression of all MRTF-A constructs showed a comparable increase in transcriptional activity, which was not further affected by coexpression of MKK6-EE (Fig. 4A). Thus, phosphorylation of MRTF-A via p38/MK2 does not influence its transactivating potential. Since the use of an overexpressed GAL4-MRTF-A construct may preclude the detection of possible weak effects of p38/MK2-mediated phosphorylation on the MRTF-A transcriptional activity, we further analysed p38/MK2-mediated transcriptional regulation of endogenous MRTF-A activity. It was previously shown, that MRTF-A is required for serum (FCS)- and cytochalasin D-mediated induction of SRF target genes^8^,^15^. Therefore, we compared the induction of MRTF target genes (SM22, Acta1) in response to FCS and cytochalasin D treatment in MK2-deficient (Fig. 4B), MK2/3 double-deficient (Fig. 4C) and MK2-rescued cell lines. To ensure a strong activation of p38/MK2 signalling and MRTF-A phosphorylation, we combined FCS and cytochalasin D treatment with anisomycin stimulation. Both FCS and cytochalasin D induce expression of selected MRTF-A target genes, which is further upregulated by addition of anisomycin, independently of MK2 (Fig. 4B,C). It was shown earlier, that TGF-beta1 induces MRTF-A binding to Smad3 what leads to activation of *slug* transcription and thereby dissociation of cell–cell contacts^16^. Therefore, we also analysed MK2/3-dependent *slug* induction by the above stimuli (Fig. 4B,C). We observed an anisomycin-dependent induction of this gene which is independent of MK2 and MK3. As a positive control, we analysed induction of the target genes SM22 and Acta1 in cells where MRTF-A was reduced by siRNA-mediated knockdown (Supplementary Figure S2). SiRNA-mediated downregulation of MRTF-A results in reduced induction of Acta1 and SM22 upon the conditions analysed. Taken together, these results support the notion that MRTF-A phosphorylation by p38/MK2 does not influence its transcriptional potential.

### p38/MK2 activation neither affects MRTF-A interaction with actin, SRF, and SMAD3 nor its homodimerisation

**Figure 5:**
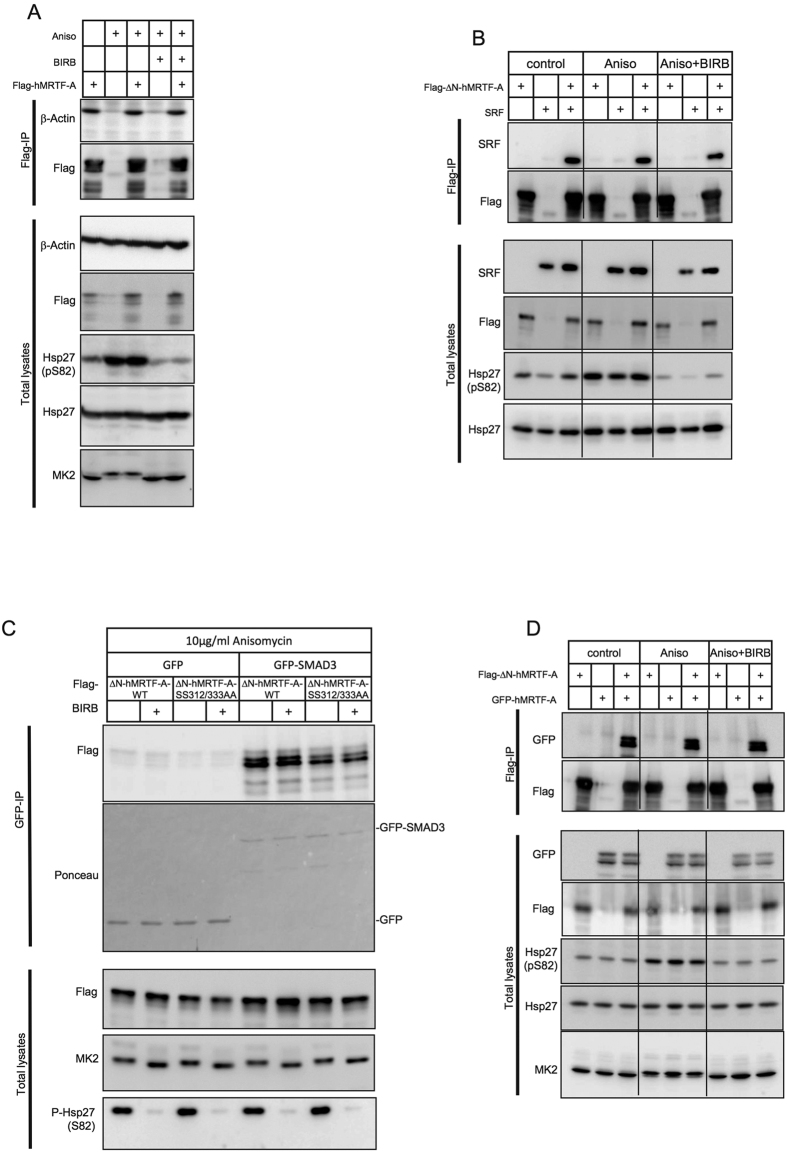
p38/MK2 activation neither affects MRTF-A interaction with actin, SRF, and SMAD3 nor its homodimerization. Serum-starved HeLa cells expressing indicated constructs were left unstimulated or were stimulated with 10 μg/ml anisomycin for 30 min with or without 30 min preincubation with 1 μM BIRB796. Extracts were analysed directly (Total lysate panels) or following immunoprecipitation with anti-flag antibody (IP panels). P38/MK2 activation does not affect any of here analyzed interactions: (**A**) Flag-hMRTF-A binding to endogenous actin. (**B**) Flag-deltaN-hMRTF-A complex formation with overexpressed SRF. (**C**) Interaction of Flag-deltaN-hMRTF-A or its SS312/333AA nonphosphorylatable mutant with GFP-SMAD3. (**D**) Flag-deltaN-hMRTF-A homodimerisation with GFP-hMRTF-A.

MRTF-A associates with non-polymerized actin through its N-terminal RPEL motifs. Activation of the Rho-actin signalling pathway induces actin polymerization, disrupts MRTF-A association with actin, promotes MRTF-A nuclear accumulation and binding to SRF thus activating target gene expression^17^. To determine whether p38/MK2-mediated phosphorylation of MRTF-A affects its binding to actin we expressed Flag-tagged hMRTF-A in HeLa cells and stimulated them with anisomycin in the presence or absence of p38^MAPK^-specific inhibitor BIRB796. The effect of p38^MAPK^/MK2 activation by anisomycin was confirmed by phosphorylation of MK2 monitored by band-shift and by the effect on phosphorylation of MK2 substrate Hsp27 at Ser^82^. As expected, the p38^MAPK^ inhibitor BIRB796 efficiently down-regulated MK2 and Hsp27 phosphorylation. In parallel we analysed the interaction of overexpressed Flag-hMRTF-A with endogenous actin by exploring actin co-precipitation with immunoprecipitated Flag-hMRTF-A by Ponceau staining and by Western blot (Fig. 5A and Supplementary Figure S3A). We could not detect any significant changes in MRTF-A/actin interaction after anisomycin-induced p38/MK2 activation or p38/MK2 inhibition (Fig. 5A). It is known, that MRTF-A binding to G-actin prevents its interaction with SRF^10^. Probably due to the great excess of co-precipitated endogenous actin, we were not able to show interaction of overexpressed SRF with Flag-hMRTF-A in co-precipitation experiments. To overcome this problem we used the Flag-ΔN-hMRTF-A mutant in which the G-actin binding RPEL domains are deleted^15^. Indeed the Flag-ΔN-hMRTF-A mutant lost the ability to interact with actin and showed detectable interaction with SRF (Supplementary Figure S3B). However, this interaction was not affected by p38^MAPK^ activation (Fig. 5B). We also analysed the role of p38^MAPK^/MK2-dependent phosphorylation of MRTF-A in complex formation with Smad3. Flag-ΔN-hMRTF-A or Flag-ΔN-hMRTF-A-SS312/333AA was co-transfected with GFP-Smad3 or GFP alone in HeLa cells. Cells were stimulated with anisomycin in the presence or absence of the p38^MAPK^ inhibitor BIRB796. Specific interaction of MRTF-A with Smad3 was independent of p38^MAPK^/MK2 activity and Ser312/Ser333 phosphorylation (Fig. 5C). We further utilized the Flag-ΔN-hMRTF mutant to analyse effects of p38^MAPK^/MK2 activation on MRTF-A/MRTF-A homodimerisation^10^. Using a co-immunoprecipitation assay, we detected a specific interaction between Flag-ΔN-hMRTF-A and GFP-hMRTF-A without any significant effect of p38^MAPK^ activation on this interaction (Fig. 5D).

### p38/MK2 activation does not affect MRTF-A intracellular localization

**Figure 6:**
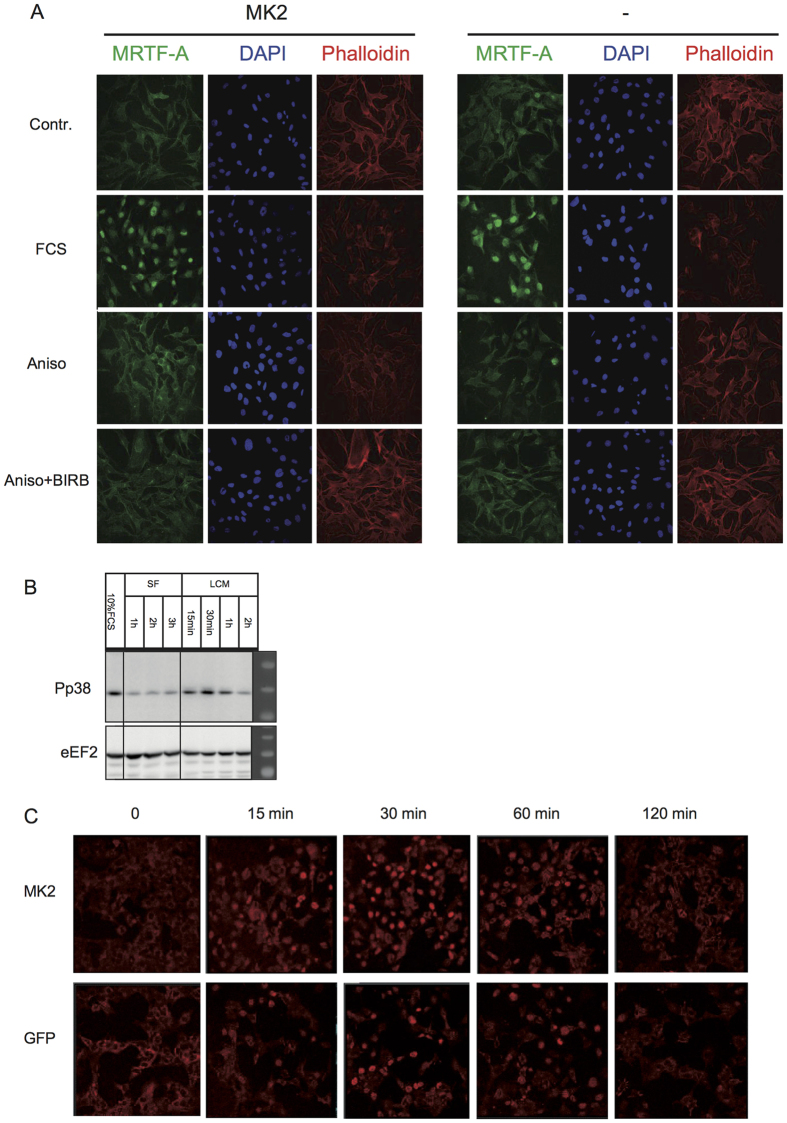
p38/MK2 activation does not affect MRTF-A intracellular localization. Subcellular localization of endogenous MRTF-A in MK2/3 DKO MEFs transduced with MK2 or empty vector was detected by immunocytochemistry and confocal microscopy. (**A**) Cells were serum starved overnight and then were left non-stimulated (Contr.) or were stimulated with 10% FCS or 10 μg/ml anisomycin for 30 min with or without preincubation with 1 μM BIRB for 30 min. The green signal denotes specific MRTF-A staining. Nuclei are stained by DAPI shown in blue and F-actin stained with Phalloidin shown in red. (**B**) Western blot analysis of low calcium medium (LCM) induced p38 phosphorylation. MEFs were starved for up to 3 hr and then were treated with LCM for the time indicated. eEF2 levels were analysed as loading controls. (**C**) MK2 does not influence LCM-induced nuclear translocation of MRTF. MK2/3 DKO MEFs rescued or not with MK2 were stimulated with LCM for the indicated times and the localization of endogenous MRTF was visualized by immunostaining.

Since MRTF-A activation is reflected by its translocation from the cytoplasm into the nucleus, we decided to analyse the influence of p38^MAPK^/MK2 activation on MRTF-A intracellular localization. Endogenous MRTF-A, which is predominantly cytoplasmic in serum starved MEFs, rapidly re-localizes to the nucleus after serum stimulation (Fig. 6A) or cytochalasin-D treatment (Supplementary Figure S4), both, in the MK2-rescued and in the MK2-deficient cells. Anisomycin treatment and p38^MAPK^ inhibition do not lead to any detectible changes in the MRTF-A intracellular distribution (Fig. 6A). Interestingly, it was shown earlier that a cell-cell-contact disruption by low-Ca^2+^ medium (LCM) activates p38^MAPK^ and triggers a nuclear accumulation of MRTF-A^18^. To investigate the MK2-dependence of this process, we first analysed the dynamics of p38^MAPK^ activation by LCM. p38^MAPK^ is rapidly but transiently activated with a maximum of phosphorylation after 30 min and a dephosphorylation back to the basal level after about 2 h (Fig. 6B). MRTF-A nuclear accumulation upon LCM correlates with p38 activation, reaching maximum after 30 min and returning back into the cytoplasm after 2 h (Fig. 6C). However, the dynamics of MRTF-A relocalisation was comparable between MK2-deficient and MK2-rescued MEF cells, indicating that MK2-mediated phosphorylation of MRTF-A does not influence this process.

### Analysis of the role of Ser312/Ser333 phosphorylation in MRTF-A target gene expression

**Figure 7:**
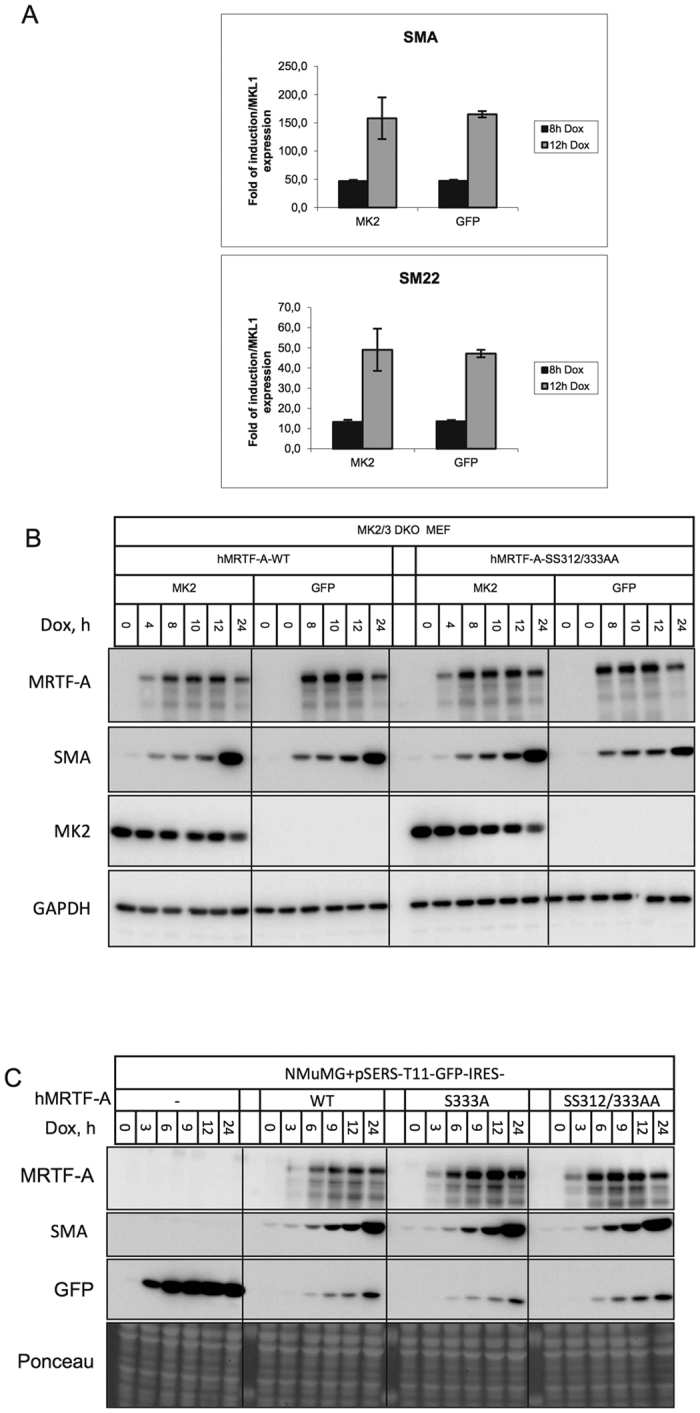
Analysis of the role of Ser312/Ser333 phosphorylation in MRTF-A target gene expression. Overexpression of MRTF-A upregulates contractile gene expression independently of MK2 and S312/S333 phosphorylation. (**A**) MK2/3 DKO MEFs stably transduced with doxycycline-inducible hMRTF-A in the presence or absence of MK2 were treated with 1 μg/ml doxycycline for 0 hr, 8 hr and 12 hr. Expression of SMA, SM22 and MRTF-A mRNA was normalized to GAPDH mRNA expression. SMA and SM22 mRNA expression is represented as fold of induction relative MRTF-A mRNA levels. (**B)** MK2/3 DKO MEFs stably transduced with doxycycline-inducible hMRTF-A or hMRTF-A-SS312/333AA nonphosphorylatable mutant in the presence or absence of MK2 were treated with 1 μg/ml doxycycline for the time indicated. Cell lysates were analyzed in Western blot with appropriate antibodies. Neither mutation of Ser312/S333 sites to Alanine nor absence of MK2 influences MRTF-A-induced SMA expression. (**C**) NMuMG cells stably transduced with retroviral TetOn hMRTF-A wild type construct or hMRTF-A-S333A or hMRTF-A-SS312/333AA mutants were treated with 1 μg/ml doxycycline for the time indicated. Cell lysates were analyzed in Western blot with appropriate antibodies. Neither mutation of Ser312/S333 sites to Alanine nor absence of MK2 influences MRTF-A-induced SMA expression. Rapid upregulation of MRTF-A expression upon doxycyclin treatment leads to strong expression of SMA, which is independent of availability of Ser312 and Ser333 to phosphorylation.

Since MRTF-A induction in the cell upregulates the expression of a variety of contractile genes^19^,^20^, we decided to analyse the role of Ser312/Ser333 phosphorylation in MRTF-A’s target gene expression in MK2/3 DKO MEFs stably transduced with the retroviral TetOn MRTF-A-WT or MRTF-A-SS312/333AA constructs and rescued or not with MK2. Doxycyclin treatment resulted in strong MRTF-A expression with robust induction of contractile genes (Fig. 7A) and strong increase of SMA protein levels (Fig. 7B) independent of MK2. Hence, upregulation of MRTF-A-regulated contractile genes upon MRTF-A induction was independent of MK2-mediated S312/S333 phosphorylation (Fig. 7A,B). Since the effect of p38/MK2-mediated phosphorylation on MRTF-dependent gene expression could be cell type specific, we also analysed whether the presence of the intact Ser312/Ser333 phosphorylation sites on MRTF-A is critical for MRTF-A-induced expression of SMA in NMuMG cell line derived from normal mouse mammary epithelial cells. A critical role of MRTF-A in epithelial–mesenchymal transition (EMT) and α-SMA expression in these cells was demonstrated earlier^16^,^21^,^22^. NMuMG cells were stably transduced with retroviral TetOn hMRTF-A wild type construct or hMRTF-A-S333A or hMRTF-A-SS312/333AA mutants. Rapid upregulation of MRTF-A expression upon doxycyclin treatment leads to strong expression of SMA, which is independent of availability of Ser312 and Ser333 to phosphorylation (Fig. 7C).

### Neither MRTF-A overexpression nor downregulation affect stress-induced IEG expression

**Figure 8:**
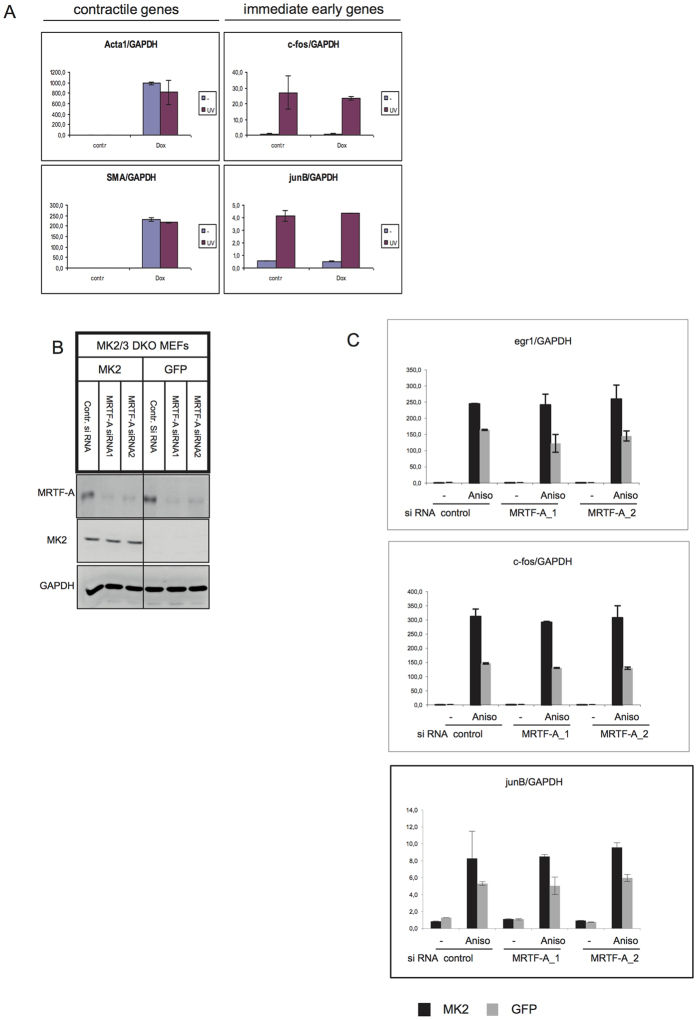
Neither MRTF-A overexpression nor downregulation affect stress-induced IEG expression. IEG expression is not influenced by upregulated or downregulated MRTF-A levels. (**A**) MEFs transduced with hMRTF-A retroviral TetOn construct were starved over night without (contr) or with 1 μg/ml Doxycyclin (Dox) and then were left unstimulated (−) or were stimulated with 200 J/qm UV-C (UV) and left to recover in serum-free medium for 1 hr. Endogenous transcript levels of Acta1, Acta2 (SMA), c-fos and junB were determined by RT–PCR and normalized to GAPDH mRNA. (**B**,**C**) MK2/3 DKO MEFs transduced with MK2 or with empty vector (GFP) were treated with two different siRNAs targeted mMRTF-A (MRTF-A siRNA) or with scrambled siRNA (Contr. siRNA). After 50 hr of transfection, cells were starved for 12 hr and then were left unstimulated (−) or were stimulated with 10 μg/ml anisomycin for 90 min (Anis) and analysed by immunoblotting (B) or by RT-PCR (C).

A role of MK2 in stress-induced transcriptional activation of immediate early genes (IEG) via SRF was shown recently^2^. To analyse whether MRTF-A overexpression interferes with stress-induced IEG expression, we used MK2/3 DKO MEFs transduced with the retroviral TetOn hMRTF-A and then rescued or not with MK2. The expression of contractile genes Acta1 and Acta2 (SMA) was strongly upregulated upon doxycycline-induced MRTF-A overexpression and was not significantly influenced by UV-stimulation (Fig. 8A). Vice versa, MRTF-A overexpression did not interfere with UV stress induced upregulation of the immediate early genes c-fos and junB (Fig. 8A). Since overexpression of MRTF-A did not interfere with IEG induction, we decided to analyse whether downregulation of MRTF-A expression, as a result of specific siRNA treatment (Fig. 8B), would have any influence on IEG induction upon stress. Previously, serum-inducible MRTF-A-dependent (junB) and MRTF-A-independent (c-fos, egr1) SRF target genes have been identified^23^. The stress induction of the IEGs junB, c-fos, and egr1 was significantly attenuated in MK2/3-knockout MEFs but was not modulated by MRTF-A knockdown (Fig. 8C). Hence, although SRF is a fundamental factor critically involved in transcriptional initiation of IEG and contractile remodelling response, its regulation in the context of specific promoters seems rather distinct for IEGs (controlled by SRF-phosphorylation via MK2 upon stress)^2^,^24^ and genes involved in contractile remodelling (controlled by MRTF-A/SRF interaction)^4^ without considerable crosstalk).

### Ser312/Ser333 phosphorylation does not influence MRTF-A induced actin polymerization

**Figure 9:**
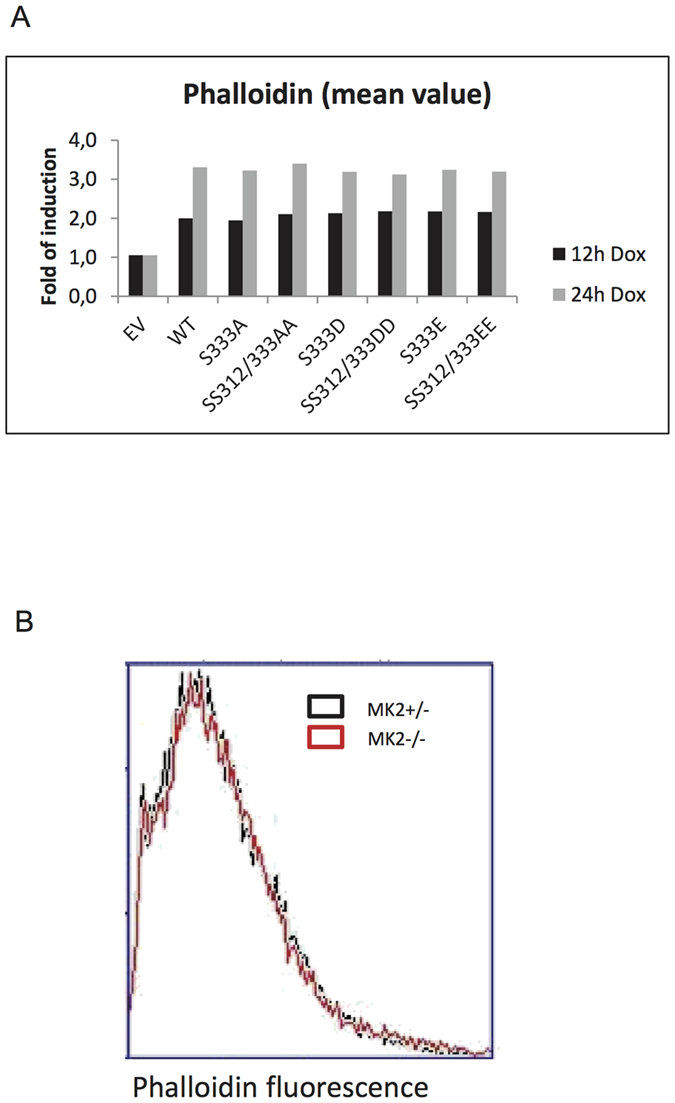
Ser312/Ser333 phosphorylation does not influence MRTF-A induced actin polymerization. **(A**,**B)** F-actin content analysis by flow cytometry **(A)** F-actin content analysed in NMuMG cells stably transduced with doxycyclin-inducible constructs expressing empty vector (EV), hMRTF-A (WT) or hMRTF-A non-phosphorylatable mutants (S333A, SS312/333AA) or hMRTF-A phospho-mimicking mutants (S333D, SS312/333DD, S333E, SS312/333EE). Upregulation of the F-actin content upon MRTF-A overexpression is independent of MRTF-A S312/S333 phosphorylation state. (**B)** Analysis of F-actin content in bone marrow derived from MK2 + /− and MK2−/− littermates.

Recently, it was shown that a frameshift mutation of MRTF-A causes a loss of filamentous actin content in lymphoid and myeloid lineage immune cells that leads to reduced phagocytosis and abrogation of cell migration^25^. Abrogation of F-actin assembly in MRTF-A-mutated cells was due to reduction of globular actin (G-actin) levels and impaired expression of multiple actin-regulating genes revealing MRTF-A as a non-redundant regulator of cytoskeleton-associated functions. Therefore, we analysed the effect of doxycyclin-induced expression of hMRTF-A and its non-phosphorylatable as well as phospho-mimicking mutants on the F-actin content in NMuMG cells. The induction of MRTF-A expression leads to a time dependent increase in the F-actin content of these cells, reaching an approximately 3-fold expression level after 24 h of induction compared to the basal level. The comparable increase in the F-actin levels is also detected for Dox-induced expression of wild type MRTF-A and the different mutants (Fig. 9A) indicating that the phosphorylation status of S312/S333 is not decisive for the regulation of the F-actin content. We also analysed the *in vivo* effect of MK2 on F-actin levels in bone marrow from MK2+ /− and MK2−/− littermates. In the case that MRTF-A phosphorylation by MK2 affects its function, we would expect differences in F-actin levels between MK2−/− and MK2+ /− bone marrow cells. However, since these cells do not display any difference in their F-actin levels as determined by FACS analysis (Fig. 9B), we conclude that MK2-dependent phosphorylation is not involved in F-actin regulation in myeloid and lymphoid cells.

## Discussion

Here, we have demonstrated a stress-dependent p38^MAPK^/MK2-driven phosphorylation of two defined, well conserved serine residues of MRTF-A in various cultured cell lines as well as *in vitro*. We followed this observation, since the modulation of MRTF-A activity by MK2-mediated phosphorylation could represent a novel crosstalk between myogenic and stress signalling. MRTF-A is a transcriptional coactivator of SRF linking actin dynamics with transcription of SRF-dependent genes encoding contractile and cytoskeletal proteins^5^,^13^,^26^. The p38^MAPK^/MK2 signalling is a general regulator of stress and inflammatory response^27^,^28^. We hypothesized that the stress-dependent phosphorylation of MRTF-A by MK2 could adapt MRTF regulation to various stress conditions. Indeed, MK2 was shown to be engaged in multiple MRTF-A-regulated cellular processes like cell migration^29^,^30^,^31^,^32^, myofibroblast differentiation^33^, fibrosis^34^ and wound healing^35^,^36^ indicating possible involvement of MK2 in direct MRTF-A regulation. The similarity of MRTF and MK2 inactivation phenotypes, like, for example, the reduced infarct size after myocardial infarction^19^,^37^,^38^, or the resistance to bleomycin induced lung fibrosis^39^,^40^ also suggests that the MK2 and MRTF-A could act in an identical genetic pathway.

Using MRTF-A phosphorylation site mutants and protein kinase inhibitors we tried to generate experimental evidence for a p38^MAPK^/MK2 phosphorylation-regulated physiological function of MRTF-A. First, we analysed the transactivating potential of MRTF-A in a classical GAL4-fusion system in transfected HeLa cells, but could not detect any changes in transactivation as a result of p38/MK2 activation by constitutive active MKK6. Next, we investigated the influence of p38/MK2-mediated phosphorylation of MRTF-A on its interaction with various relevant intracellular components as well as its dimerization properties by immunoprecipitation of Flag-tagged MRTF-A from lysates of transfected HeLa cells. Neither activation nor downregulation of p38/MK2 signalling or mutation of MRTF-A phosphorylation sites affects MRTF-A interaction with actin, SRF, SMAD3 or MRTF-A homo-dimerization. Further analysis of ectopic overexpression of MRTF-A or MRTF-A-downregulation in MEFs revealed that neither MRTF-A overexpression nor its downregulation interfere with MK2-dependent stress-induced IEG expression. Induction of “contractile” gene expression in response to MRTF-A overexpression in MEFs was also independent of MK2. In addition, we analysed the role of MRTF-A phosphorylation in SMA expression and actin remodelling in NMuMG cells. The role of MRTF-A in the switch between epithelial and mesenchymal markers (epithelial mesenchymal transition) is well established in this cell line. However, expression of wild type and phosphorylation mutants of MRTF-A results in comparable increase of SMA expression and F-actin levels in these cells. Since it could not be excluded that the properties of overexpressed fusion-proteins do not completely match the properties of the endogenous MRTF-A, we analysed the effect of stress on endogenous MRTF-A intracellular localization using immunostaining with MRTF-A specific antibodies. We found that serum stimulation, cytochalasin treatment and calcium deprivation lead to rapid nuclear translocation of MRTF-A, but independent of MK2. Even anisomycin treatment, which highly activates p38/MK2 and leads to strong MRTF-A phosphorylation, did not affect MRTF-A intracellular localization.

Hence, using various approaches, we could not identify MK2-dependent properties or functions of MRTF-A. It could well be, that the overexpression of MRTF-A used in most experiments precludes the detection of a fine-tuning of the MRTF-A function. It could also be that MK2-mediated phosphorylation of MRTF-A is relevant in a specific cellular context or cell type. Additionally, we demonstrated direct MRTF-A phosphorylation by p38^MAPK^ on non-identified site/s. Hence, we cannot completely exclude that a functional relevant stress-mediated MRTF-A phosphorylation requires a concerted phosphorylation of the MK2 and the p38^MAPK^ site/s together. To assign a clear function to the MK2- and p38^MAPK^-dependent phosphorylation of MRTF-A, the generation of “knockin” mice lacking the various phosphorylation sites of MRTF-A would be helpful in the future.

An unlikely explanation of our results could be the existence of non-functional protein phosphorylation sites in MRTF-A, which have been recently postulated for many other proteins^41^. This idea of non-functional protein phosphorylations especially arose from the enormous number of phosphorylated amino acid side chains identified in mass spectrometric analyses of proteins and proteomes. Since normal mass spectroscopy does not provide direct quantitative data, no information on the stoichiometry of protein phosphorylation is obtained and some of the phosphorylations identified could be minor non-functional “cross-phosphorylations”, which influence only a tiny fraction of a specific protein population. However, the detection of the MRTF-A phosphorylations using phospho-site specific antibodies in Western blot analysis demonstrated higher amounts of MRTF-A phosphorylated than that detectable by sensitive mass spectrometry, making a minor “cross-phosphorylation” of MRTF-A by MK2 rather unlikely. Nevertheless, there could be some cases where even stoichiometric phosphorylations of a protein do not change its function. In the rare case that a phosphorylatable amino acid at a specific position is relevant for the assembly of the protein’s 3D-structure and its phosphorylation later does not change the relevant protein properties, such non-functional phosphorylation sites could even be evolutionary conserved. However, such case would be rather uncommon and the evolutionary conservation of the MK2-phosphorylation sites of MRTF-A in various, evolutionary distant vertebrates (Fig. 1C) makes a non-functional phosphorylation at these sites at least unlikely. At the end, the proof that a specific phosphorylation of a protein is non-functional is difficult. Moreover, in recent years an increasing number of non-canonical “moonlighting” activities of specific proteins, and even well established metabolic enzymes, have been described in eukaryotic cells^42^. Hence, a specific phosphorylation irrelevant for the canonical function of a protein, such as MRTF-A, might well be relevant for its so far undiscovered non-canonical activity.

## Methods

### Antibodies and reagents

p38 MAPK, phospho-p38 MAPK, phospho-HspB1 (mouse-S86/human-S82), MK-2, phospho-MK-2 (T222), and P-(S/T)-PKD Substrate antibodies were from Cell Signalling Technology. Anti-GAPDH antibody was from Millipore, anti-SMA from Sigma, anti-Flag from Stratagene. Anti-Hsp27 (C20), -eEF2 (C14), -H3 (FL-136), -GFP (B2), -MRTF-A (C19), -SRF (G-20), -Actin (C-4), and horseradish peroxidase-conjugated secondary antibodies were from Santa Cruz Biotechnology. Anisomycin, cytohalasin D (CytD) and latrunculin B were from Calbiochem, SB202190 and BIRB-796 from Axon Ligands. Control (Allstars negative control) and mMRTF-A-siRNAs (Mm_Mkl1_2: 5′-CCCACTCAGGTTCTTTCTCAA-3′; Mm_Mkl1_4: 5′-CGAGGACTATTTGAAACGGAA -3′), and HiPerFect transfection reagent were obtained from Qiagen. Power SYBR Green Cell-to-CT Kit was from Ambion. PD184352 was from Upstate Biotechnology. Arsenite, Cycloheximide (CHX), Doxycyclin (Dox), Polyethylenimine (PEI) and Phalloidin-TRITC were from Sigma. 2xSensiFAST SYBR No-ROX Mix was from Bioline. Primers for junB amplification were from Qiagen (QT00241892).

### Cells culture and treatments

All cells were cultured in DMEM (GIBCO, Cat. 41966) supplemented with 10% FCS (PAA), 100 U of penicillin G/ml and 100 mg of streptomycin/ml (PAA) under humidified conditions with 5% CO2 at 37 °C. MK2- and MK2/3- deficient immortalized MEFs and macrophages rescued with MK2, MK2-K79R or control vector by retroviral transduction were generated as reported earlier^43^,^44^. HeLa and HEK 293T cells were transfected using Polyethylenimine (PEI): 4 × 10^6^ cells were seeded in 10 ml of growth medium in a 10 cm plate. On the next day medium was replaced by 5 ml of DMEM supplemented with 10% FCS, without antibiotics. 10 μg DNA was mixed with 0,5 ml Opti-MEM, followed by addition of 40 μl of 1 μg/μl PEI and incubation for 15 min at room temperature. The mixture was then added to the cells. After 8 hr of incubation medium was replaced with complete growth medium and cells were further cultivated for the next 24–36 hr before stimulation and/or harvesting. siRNA transfection of MEF cells was performed using HiPerFect reagent (Qiagen): shortly before transfection 5 × 10^3^ cells were seeded per well in 96-well plate in 150 μl of complete growth medium. To transfect one well 0,2 μl of 125 ng/μl siRNA was diluted in 50 μl serum-free culture medium followed by addition of 0,75 μl of HiPerFect transfection reagent and subsequent incubation of the mixture for 5–10 min at room temperature. The mixture was then added to the cells and after 50 hr of incubation the medium was replaced by serum-free medium for the next 12 hr before stimulation with 10 μg/ml anisomycin for 90 min and subsequent RNA extraction using Cell-to-CT Kit (Ambion). MEF cells were transfected by electroporation using MicroPorator (Peqlab). 450 μl of 3 × 10^7^ cells/ml in serum free medium were mixed with 45 μg Plasmid and electroporated in 100 μl tips at 1680 V (pulse voltage), 20 ms (pulse width), 01 pulse (pulse number). 6 × 10^6^ electroporated cells were seeded into 10 cm plate in 5 ml of serum-free and antibiotic-free medium. After 1 hr of incubation 10 ml of complete medium was added for the next 16 hr before treatment. Low calcium medium treatment (LCM) proceeds as follow: cells were washed once with PBS prior to stimulation and then were treated with calcium-free DMEM from GIBCO (Cat. 21068) supplemented with L-Glutamine, Pyruvate, penicillin, streptomycin for the time indicated. Control cells were treated on the same way, but the medium was additionally supplemented with 2 mM CaCl.

### Plasmids and cloning

Gal4-BD-mMRTF-A construct was a gift from E. Olson. Plasmid 11978 (p3xFlag-hMKL1) and plasmid 27176 (p3XFLAG-hMKL1-~100) were from Addgene. pcDNA3-SRF was a gift from Michael Greenberg (Harvard Medical School, Cambridge). Human MRTF-A coding region was PCR amplified from p3xFlag-MKL1 plasmid (fwd primer-5′- CACCATGCCGCCTTTGAAAAGTCC-3′ and rev primer- 5′-CTACAAGCAGGAATCCCAGTGCAG-3′) and cloned into pENTR-D-TOPO vector (Invitrogen) following the manufacturers protocol. Destination expression vectors with GFP tag was generated by LR recombination reactions (Invitrogen). Site directed mutagenesis was performed using Quickchange mutagenesis kit (Stratagene). Retroviral bidirectional tetracycline-inducible all-in-one vector, integrating all components required for tet-regulated transgene expression (Heinz *et al*. 2011) was modified so that full-length hMRTF-A was subcloned upstream of the internal ribosome entry site (IRES) and the eGFP was positioned downstream of the IRES: pSERS-T11-hMRTF-A-IRES-GFP-PGK-M2. The construction of such bicistronic vector encoding the gene of interest and eGFP as a marker allows sorting for effectively transduced, eGFP positive cells by FACS. The pcDNA3-Flag-MKK6-EE and the bicistronic pMMP-IRES-GFP retroviral vectors expressing MK2 or MK2 catalytic dead mutant (K79R) were reported earlier^43^,^45^.

### Retroviral gene transfer

MK2/MK3 double-deficient immortalized mouse embryonic fibroblasts or macrophages were transduced with the bicistronic pMMP-IRES-MK2, -MK2-K79R (KR) or empty (GFP) vectors following the previously reported method^43^. To achieve controlled expression of MRTF-A, the MK2/3 KO MEFs were firstly transduced with doxycyclin (Dox)-inducible vector coding for hMRTF-A (pSERS-T11-hMRTF-A-IRES-GFP-PGK-M2) or with empty vector (EV). The resulted cell lines were treated overnight with 1 μg/ml Dox to induce GFP expression and then cells were sorted by FACS for comparable expression of GFP. The sorted cells were used then for the further transduction with the bicistronic pMMP-IRES-MK2, or empty (GFP) vectors following earlier described procedure^43^.

### Real-time PCR

MEFs were stimulated as indicated and RNA was extracted using the NucleoSpin RNA purification method (Machery-Nagel) and reverse-transcribed (Fermentas). Alternatively Cell-to-CT method of RNA extraction and reverse transcription (Ambion) was used following manufacture protocol. Real-time PCR (Q-PCR) was carried out in Rotor-Gene Q (Qiagen) using SYBR Green chemistry (2xSensiFAST SYBR No-ROX Mix (Bioline)) for Acta1, Acta2 (SMA), Tagln. (SM22), slug, egr1, c-fos and junB transcripts. PCR was performed as follows 95 °C for 2 min, then 45 cycles of 95 °C for 5 s, 60 °C for 15 s; and 60 °C for 1 min. The threshold cycle (CT) for each individual PCR product was calculated by the instrument software, and CT values obtained were normalized against GAPDH mRNA. Detailed primer sequences are listed in the Supplementary Table S5.

### Immunoprecipitation

For Flag-IP HEK293T or HeLa cells were transfected with indicated expression constructs and 24 hrs post-transfection cells were lysed in Flag-IP-lysis buffer (50 mM Tris-pH 7.4, 150 mM NaCl, 1 mM EDTA, 10 mM beta-glycerophosphate, 1% TX-100, Roche protease inhibitor cocktail, Sigma phosphatase inhibitor cocktail 3). Supernatants were incubated with Anti-Flag-M2 Affinity Gel (Sigma) for ~5 hr at 40 °C. Beads were then washed 5 times with IP-Buffer (1xTBS, 50 mM NaF, 1% Triton X-100, 1 mM Na3VO4), boiled in gel loading buffer, and analysed in western blot. GFP-immunoprecipitation with GFP nanobodies was carried out as described earlier^46^.

### *In vitro* kinase assay

Flag-hMRTF-A was immuno-affinity purified from HEK293T cells as described in “Immunoprecipitation” section, except that, the phosphatase inhibitor cocktail and beta-Glycerophosphate were excluded from the Flag-IP-lysis buffer. After 2 washes with IP-Buffer, beads were additionally washed 3 times with 1xNEB restriction buffer and treated with 20 U of CIP alkaline phosphatase (NEB Biolabs) for 20 min at 37 °C. After CIP treatment resin was additionally washed 5 times with IP-Buffer and then heated at 90 °C for 2 min to inactivated kinases, which could be possibly co-precipitated from the cell lysates. The resulted beads with immobilized Flag-MKL1 were used as a substrate in *in vitro* kinase assay with ~1 μg recombinant bacterially purified GST-MK2 or GST-p38alpha or both together in buffer containing 50 mM Na-beta-glycerophosphate, 0,1 mM EDTA, pH 7.4, 4 mM MgAc, 1 μCi [γ-33P]-ATP. Kinase reaction was carried out in 25 μl final volume at 30 °C for 30 min. Reaction was stopped by adding 8 μl 4x Laemmli buffer (0.25 M Tris/HCl pH 6.8, 4% (m/v) SDS, 4 mM EDTA, 0.25% (m/v) Bromophenol Blue, 20% β-Mercaptoethanol, 40% Glycerol) and samples were heated to 96 °C for 5 min. Protein samples were separated on a 7.5–22.5% polyacrylamide gradient gel. After separation, proteins were fixed in the gel using a coomassie staining-solution (250 mg/L Coomassie G, 40% methanol, 10% glacial acetic acid). After destaining the appearing proteins bands were analysed using the Image Analyzer LAS-3000 (Fujifilm). The gel was dried for 2 hr at 70 °C using a gel dryer. Radioactivity incorporated into substrates was quantified by phosphoimaging using a FLA9000 (Fuji).

### Western blotting

Soluble protein extract was run on sodium dodecyl sulfate–7.5–22.5% polyacrylamide gradient gel electrophoresis and transferred to Hybond ECL membranes (Amersham Pharmacia Biotech). Blots were incubated for 30 min in PBS–1% Tween 20 containing 5% powdered skim milk. After blocking membranes were incubated for 16 h with the primary antibody at 4 °C and for 1 h with horseradish peroxidase-conjugated secondary antibodies (diluted 2,000-fold) at room temperature. Blots were developed with self-made ECL detection kit (solution A: 1,2 mM luminol in 0,1 M Tris-HCl (pH 8.6); solution B: 6,7 mM p-coumaric acid in DMSO; solution C: 35% H2O2; ratio A : B : C is 3333 : 333 : 1), and the digital chemiluminescence images were taken by a Luminescent Image Analyser LAS-3000 (Fujifilm).

### Immunocytochemistry

MEFs were seeded on poly-l-lysine-coated coverslips, serum starved for 22 hr and then left unstimulated or were stimulated with 10% FCS, 2 μM cytochalasin D or 10 μg/ml anisomycin for 30 min. Where indicated, 1 μM BIRB was added to the cells 30 min prior to stimulation. For low calcium treatment cells were starved for 6 hr, washed once with PBS and then incubated in LCM for the time indicated. After treatment cells were washed once with PBS, fixed with 4% paraformaldehyde for 10 min at room temperature and then permeabilised with 0.2% Triton for 5 min. After 1 hr of incubation with 10% heat inactivated Horse Serum (iHS) in PBS, cells were incubated for 1 h with anti-MRTF-A (C-19, sc-21558) primary antibodies (1:200 diluted with 5% iHS/PBS). After three PBS washes, secondary antibodies - Cy3 donkey anti-goat (705–165–003 Jackson ImmunoResearch) together with Phalloidin (Alexa Fluor 647 A-22287) antibodies (1:500 diluted with 5% iHS/PBS) were added and incubated for 1 hr. After 0,5 μg/ml DAPI (ROTH) staining for 5 min coverslips were three times washed with PBS and then mounted with a help of Roti-Mount Fluor Care (ROTH) and analysed using a Leica DM IRBE microscope (40× oil immersion objective) with the Leica TCS confocal systems program.

### Luciferase reporter assay

HeLa cells were seeded 105cells/well in 24 well plates and were transfected on the next day using polyethylenimine reagent. The plasmid ratio was follow: 100 ng GFP (to control transfection efficiency), 100 ng Renilla (internal standard), 400 ng Luciferase reporter, and 20 ng activator plasmid (Gal4-BD-MRTF-A and its derivates). Where indicated 100 ng of MKK6-EE was added. The total amount of DNA per well was kept constant by adding pcDNA3-flag expression vector. Twenty four hours posttransfection cells were lysed and luciferase activity was measured using Glomax (Promega).

### Phosphoproteomics

For a phospho-proteomics approach we used MK2/3-deficient immortalized mouse embryonic fibroblasts (MEF) or macrophages (MF) reconstituted or not with MK2^43^,^44^. Cells were stress-stimulated for 30 min with 10 μg/ml anisomycin (MEF) or 1 μg/ml LPS (MF). Lysates were digested by GluC protease, and the resulting peptides were immuno-affinity purified by a PKD phosphorylation motif-specific antibody (anti-LXRXXpS/pT). The purified peptides were analysed by mass spectrometry (PhosphoScan, Cell signaling).

### FACS analysis of actin polymerization

NMuMG cells were transduced with approximately 50% efficiency with retroviral expressing TetOn constructs pSERS-T11-GFP-IRES-empty vector or vector expressing MRTF-A-WT or –S333A or -SS312/333AA or –S333D or -SS312/333DD or –S333E or -SS312/333EE mutants. 4 × 10^5^ transduced cells were seeded on 6 cm plates in triplicates. Two days later cells reached about 95% confluence and were treated or not with 1 μg/ml doxycyclin for 12 or 24 hr. Cells were collected by trypsination, washed once with PBS, fixed with 4% PFA for 15 min at room temperature, washed once with PBS, permeabilised with 0,1% Triton-X100 for 5 min, washed once with PBS, blocked with 1% BSA and stained with Phalloidin-Alexa Fluor 647 (Molecular Probes A-22287; 1:200 dilution in 1% BSA in PBS) for 30 min, distributed in 300 μl PBS and analysed by FACS. Not transduced population of the cells (~50% of GFP negative cells) was used as internal control in each separate sample. Mean value of phalloidin staining intensity of GFP positive cells was normalized to the mean value of phalloidin staining intensity of GFP negative cells and performed as fold of induction relative mean value of phalloidin staining of empty vector transduced cells.

Bone marrow was isolated after standard protocol by flashing out the mice femurs with serum-free DMEM supplemented with 100 U of penicillin G/ml and 100 mg of streptomycin/ml. Each preparation of bone marrow was divided into two parts. One part was stained with 1 μM carboxyfluorescein diacetate succinimidyl ester (CFSE (eBioscience)) in serum-free medium for 10 min at 37 °C and the second part remained unstained. After two washes in serum-free medium the stained bone marrow of MK2+ /− was combined with unstained bone marrow of MK2−/− and vice versa, followed by PFA-fixation, triton permeabilisation, phalloidin staining and FACS analysis as described above.

## Additional Information

**How to cite this article**: Ronkina, N. *et al*. Stress-dependent phosphorylation of myocardin-related transcription factor A (MRTF-A) by the p38^MAPK^/MK2 axis. *Sci. Rep.* **6**, 31219; doi: 10.1038/srep31219 (2016).

## Supplementary Material

Supplementary Figure S1

Supplementary Figure S2

Supplementary Figure S3

Supplementary Figure S4

Supplementary Table S5
