# Supplementary material for: Stress-dependent phosphorylation of myocardin-related transcription factor A (MRTF-A) by the p38MAPK/MK2 axis
Source: Sci Rep. 2016 Aug 5;6:31219. doi: 10.1038/srep31219 (PMC4974569; doi:10.1038/srep31219)
Supplement: Supplementary Figure S1 [file srep31219-s1.pdf]

Stress-dependent phosphorylation of myocardin-related transcription factor A (MRTF-A) by the p38^{MAPK}/MK2 axis

by

Natalia Ronkina, Juri Lafera, Alexey Kotlyarov and Matthias Gaestel*

Department of Biochemistry, Hannover Medical School, Hannover, Germany,

*Corresponding author

E-mail: gaestel.matthias@mh-hannover.de

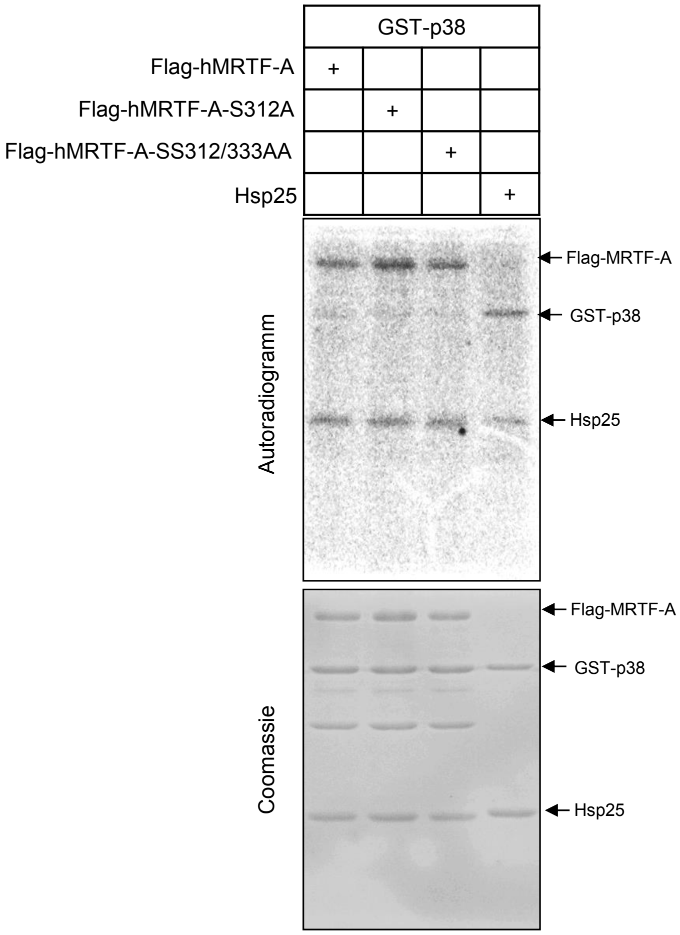

S1. In vitro phosphorylation of MRTF-A by p38^{MAPK}.

Purified Flag-hMRTF-A and its MK2-phosphorylation site mutants were incubated with recombinant GST-p38 in an invitro kinase assay. Wild type and mutant proteins were phosphorylated by p38^{MAPK} to a comparable degree.
