# Supplementary material for: Stress-dependent phosphorylation of myocardin-related transcription factor A (MRTF-A) by the p38MAPK/MK2 axis
Source: Sci Rep. 2016 Aug 5;6:31219. doi: 10.1038/srep31219 (PMC4974569; doi:10.1038/srep31219)
Supplement: Supplementary Figure S2 [file srep31219-s2.pdf]

Stress-dependent phosphorylation of myocardin-related transcription factor A (MRTF-A) by the p38^{MAPK}/MK2 axis

by

Natalia Ronkina, Juri Lafera, Alexey Kotlyarov and Matthias Gaestel*

Department of Biochemistry, Hannover Medical School, Hannover, Germany,

*Corresponding author

E-mail: gaestel.matthias@mh-hannover.de

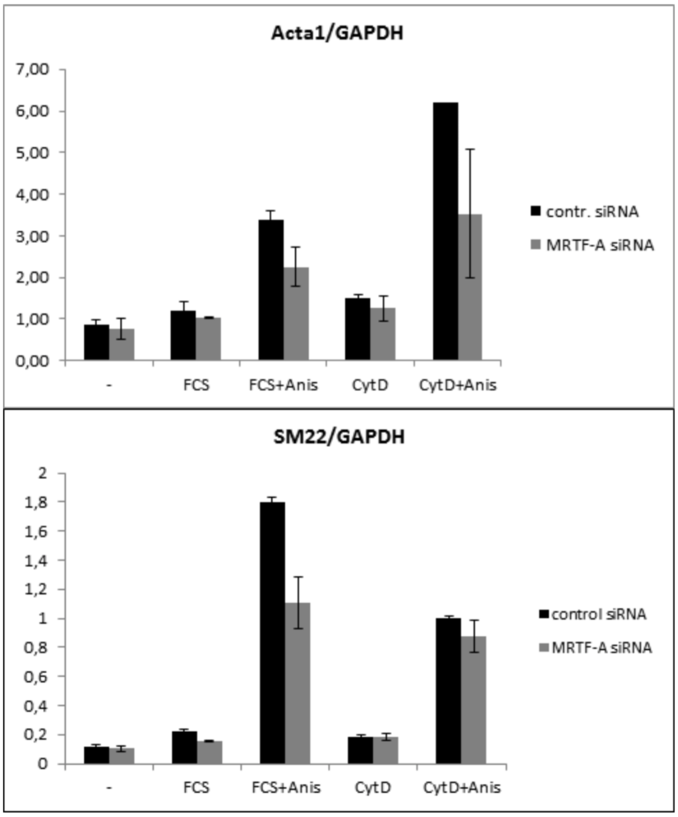

S2. MRTF-A knockdown reduces induction of the target genes Acta 1 and SM22.

Effect of serum or cytochalasin D or their combination with anisomycin on MRTF-A target gene expression in MEFs transfected with siRNA against MRTF-A or scrambled control siRNA. After 48 h cells were starved overnight and then were left unstimulated or were stimulated for 90 min with 2 µM cytochalasin D (CytD) or 10% FCS with or without their combination with 10 µg/ml anisomycin. Relative mRNA levels for acta1 and SM22 were measured by quantitative real-time PCR using the SYBR green method.
