# Supplementary material for: Stress-dependent phosphorylation of myocardin-related transcription factor A (MRTF-A) by the p38MAPK/MK2 axis
Source: Sci Rep. 2016 Aug 5;6:31219. doi: 10.1038/srep31219 (PMC4974569; doi:10.1038/srep31219)
Supplement: Supplementary Figure S3 [file srep31219-s3.pdf]

Stress-dependent phosphorylation of myocardin-related transcription factor A (MRTF-A) by the p38^{MAPK}/MK2 axis

by

Natalia Ronkina, Juri Lafera, Alexey Kotlyarov and Matthias Gaestel*

Department of Biochemistry, Hannover Medical School, Hannover, Germany,

*Corresponding author

E-mail: gaestel.matthias@mh-hannover.de

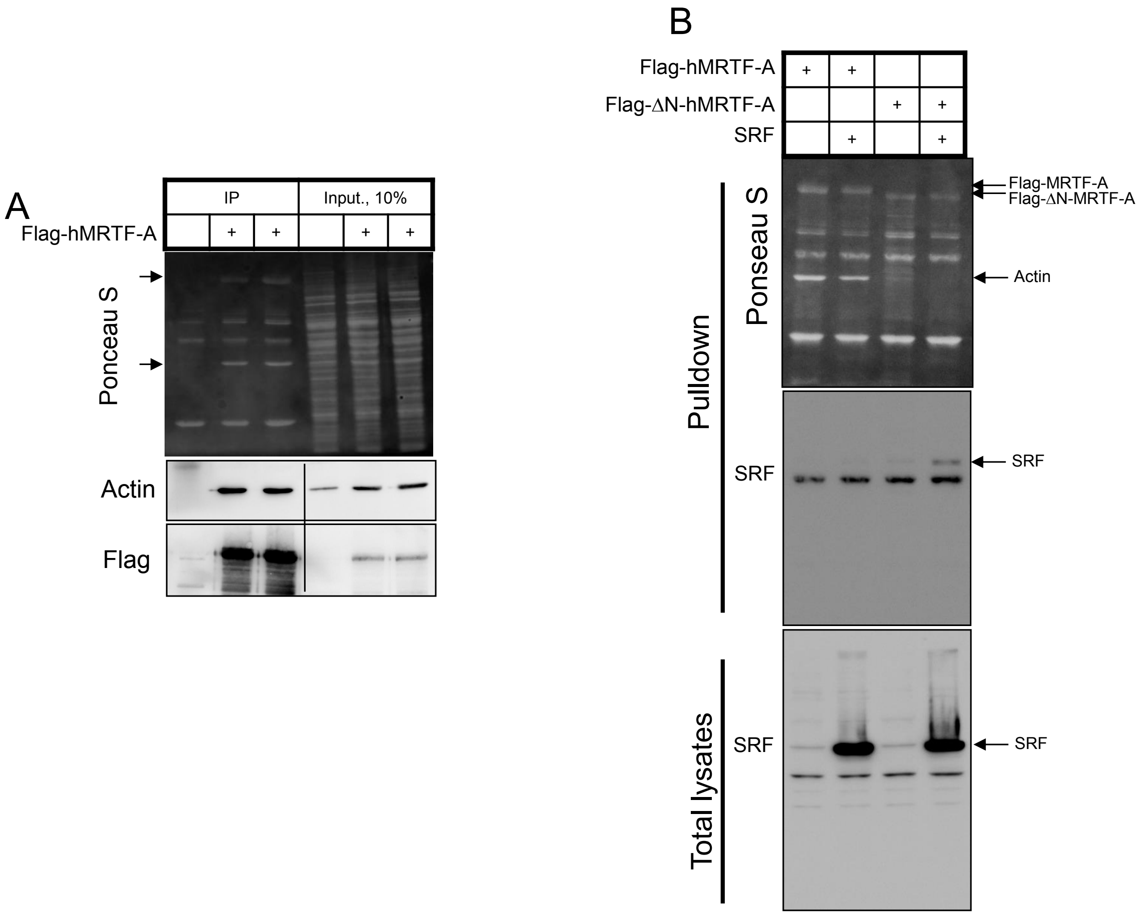

S3. Flag-deltaN-MRTF-A loses the ability to bind to endogenous actin and gains the ability to bind to SRF.

A. Flag-hMRTF-A overexpressed in HeLa cells co-precipitates endogenous actin in anti-Flag immunoprecipitation experiment. B. Flag-deltaN-hMRTF-A mutant loses the ability to interact with endogenous actin and shows interaction with overexpressed SRF.
