# Supplementary material for: Stress-dependent phosphorylation of myocardin-related transcription factor A (MRTF-A) by the p38MAPK/MK2 axis
Source: Sci Rep. 2016 Aug 5;6:31219. doi: 10.1038/srep31219 (PMC4974569; doi:10.1038/srep31219)
Supplement: Supplementary Figure S4 [file srep31219-s4.pdf]

# Stress-dependent phosphorylation of myocardin-related transcription factor A (MRTF-A) by the p38$^{MAPK}$/MK2 axis

by

Natalia Ronkina, Juri Lafera, Alexey Kotlyarov and Matthias Gaestel*

Department of Biochemistry, Hannover Medical School, Hannover, Germany,

*Corresponding author

E-mail: gaestel.matthias@mh-hannover.de

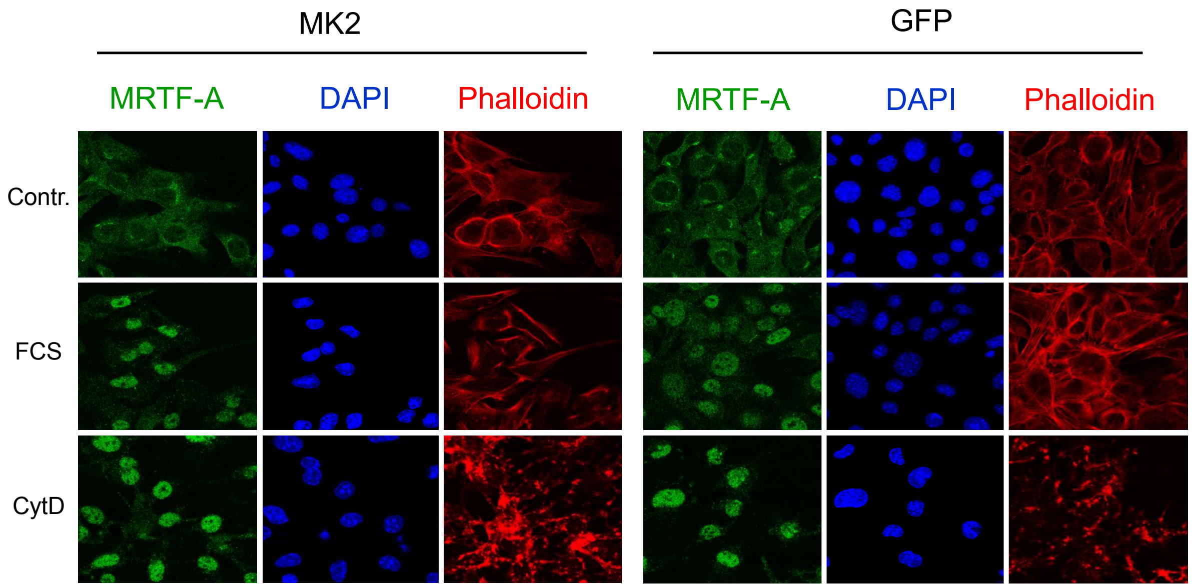


***S4. FCS- and Cytochalasin D-induced MRTF-A nuclear accumulation is not MK2-dependent.*** Subcellular localization of endogenous MRTF-A in MK2/3 DKO MEFs transduced with MK2 or empty vector (GFP) was detected by immunocytochemistry and confocal microscopy. Cells were serum starved overnight and then were left non-stimulated (Contr.) or were stimulated with 10% FCS or 2µM Cytochalasin D for 1hr. The green signal denotes specific MRTF-A staining. Nuclei are stained by DAPI shown in blue and F-actin stained with Phalloidin shown in red.
