# Supplementary material for: Stress-dependent phosphorylation of myocardin-related transcription factor A (MRTF-A) by the p38MAPK/MK2 axis
Source: Sci Rep. 2016 Aug 5;6:31219. doi: 10.1038/srep31219 (PMC4974569; doi:10.1038/srep31219)
Supplement: Supplementary Table S5 [file srep31219-s5.pdf]

Stress-dependent phosphorylation of myocardin-related transcription factor A (MRTF-A) by the p38^{MAPK}/MK2 axis

by

Natalia Ronkina, Juri Lafera, Alexey Kotlyarov and Matthias Gaestel*

Department of Biochemistry, Hannover Medical School, Hannover, Germany,

*Corresponding author

E-mail: gaestel.matthias@mh-hannover.de

Gene	Sense	Antisense
Acta1	AATGAGCGTTTCCGTTGC	ATCCCCGCAGACTCCATAC
Acta2 (SMA)	CCCACCCAGAGTGGAGAA	ACATAGCTGGAGCAGCGTCT
Tagln. (SM22)	CCTTCCAGTCCACAAACGAC	GTAGGATGGACCCTTGTTGG
slug	ATGCCGCGCTCCTTCCTGG	GTGTGAGTTCTAATGTGTCCTTGAAG
egr1	ACAGAAGGACAAGAAAGCAGAC	CCAGGAGAGGAGTAGGAAGTG
c-fos	CTACTGTGTTCCTGGCAATAGC	AACATTGACGCTGAAGGACTAC
GAPDH	CATGGCCTTCCGTGTTCCTA	CCTGCTTCACCACCTTCTTGAT

S5. Primer sequences.
